# Development of a cell-free split-luciferase biochemical assay as a tool for screening for inhibitors of challenging protein-protein interaction targets

**DOI:** 10.12688/wellcomeopenres.15675.1

**Published:** 2020-02-06

**Authors:** Rachel Cooley, Neesha Kara, Ning Sze Hui, Jonathan Tart, Chloë Roustan, Roger George, David C. Hancock, Brock F. Binkowski, Keith V. Wood, Mohamed Ismail, Julian Downward

**Affiliations:** 1 Francis Crick Institute, London, UK; 2 AstraZeneca, Cambridge, UK; 3 Promega, Madison, Wisconsin, USA; 4 Institute of Cancer Research, UK, London, UK

**Keywords:** KRAS, PI3K, RAF, protein-protein interaction, luciferase, screening

## Abstract

Targeting the interaction of proteins with weak binding affinities or low solubility represents a particular challenge for drug screening. The NanoLuc ^â ^® Binary Technology (NanoBiT ^â ^®) was originally developed to detect protein-protein interactions in live mammalian cells. Here we report the successful translation of the NanoBit cellular assay into a biochemical, cell-free format using mammalian cell lysates. We show that the assay is suitable for the detection of both strong and weak protein interactions such as those involving the binding of RAS oncoproteins to either RAF or phosphoinositide 3-kinase (PI3K) effectors respectively, and that it is also effective for the study of poorly soluble protein domains such as the RAS binding domain of PI3K. Furthermore, the RAS interaction assay is sensitive and responds to both strong and weak RAS inhibitors. Our data show that the assay is robust, reproducible, cost-effective, and can be adapted for small and large-scale screening approaches. The NanoBit Biochemical Assay offers an attractive tool for drug screening against challenging protein-protein interaction targets, including the interaction of RAS with PI3K.

## Introduction

While enzymatic activities have generally been the preferred starting point for the development of drugs targeting biological processes, there are not always suitable tractable enzymatic targets in signalling pathways of interest. In such cases, the transfer of biological signals is likely to be also dependent on specific protein-protein interactions (PPIs), which can make attractive alternative targets for drug discovery. The selection of a suitable binding assay is a key factor in the development of screens to identify inhibitors of protein-protein interactions. The selection process for a cell-free, or biochemical, assay will depend mainly on the nature of the protein complex under investigation, in particular the binding affinities and solubilities of the components. Protein complexes with low binding affinity pose a particular challenge for drug screening assays for a number of reasons. First, for optimal biochemical assay screening, one of the protein concentrations should be close to the K _d_ (dissociation constant) of the complex binding affinity. At this concentration, the protein will exist in a 50% unbound and 50% complexed state, which, in the screening process, allows for detection of weakly binding compounds that disrupt the protein interaction of interest. Protein complexes with weak binding affinities (low micromolar and above) thus require large amounts of proteins for drug screening, which is not always feasible, especially for poorly soluble proteins. Second, weak binding protein complexes require more screening reagents when using biochemical assays such as HTRF (Homogeneous Time Resolved Fluorescence) which may be prohibitively expensive for high throughput screening ^1, 2^.

The RAS family of oncoproteins (HRAS, NRAS and KRAS) are key regulators of cellular proliferation and when mutationally activated, as in many cancers, can drive tumor formation. They cycle between inactive (RAS-GDP bound) and active (RAS-GTP bound) forms, with the latter contributing to a wide range of cellular signaling through direct interaction with effector enzymes such as RAF protein kinase isoforms (ARAF, BRAF and CRAF/RAF1) and members of the PI3K family of lipid kinases (p110α, δ and γ) through their RAS binding domains (RBDs), resulting in the activation of the MAPK and PI3K signaling pathways ^3– 7^. Oncogenic mutations in RAS lock it into the GTP-bound form, causing the constant activation of downstream pathways thereby contributing to the genesis of several commonly occurring types of cancer ^6, 8, 9^. Therefore, finding inhibitors that can block the RAS/RAF and/or the RAS/PI3K interaction would be a significant development toward future cancer therapies ^10– 12^. However, a significant stumbling block in the quest to inhibit these protein complexes is the fact that RAS interacts with RAF and PI3K with two widely differing affinities (K _d_ ∼ 20 nM and 3 µM respectively) ^13– 15^. The relatively weak interaction between RAS and PI3K makes this a challenging protein complex for the selection of a suitable biochemical assay for drug screening for the reason addressed above.

**Figure 1.**
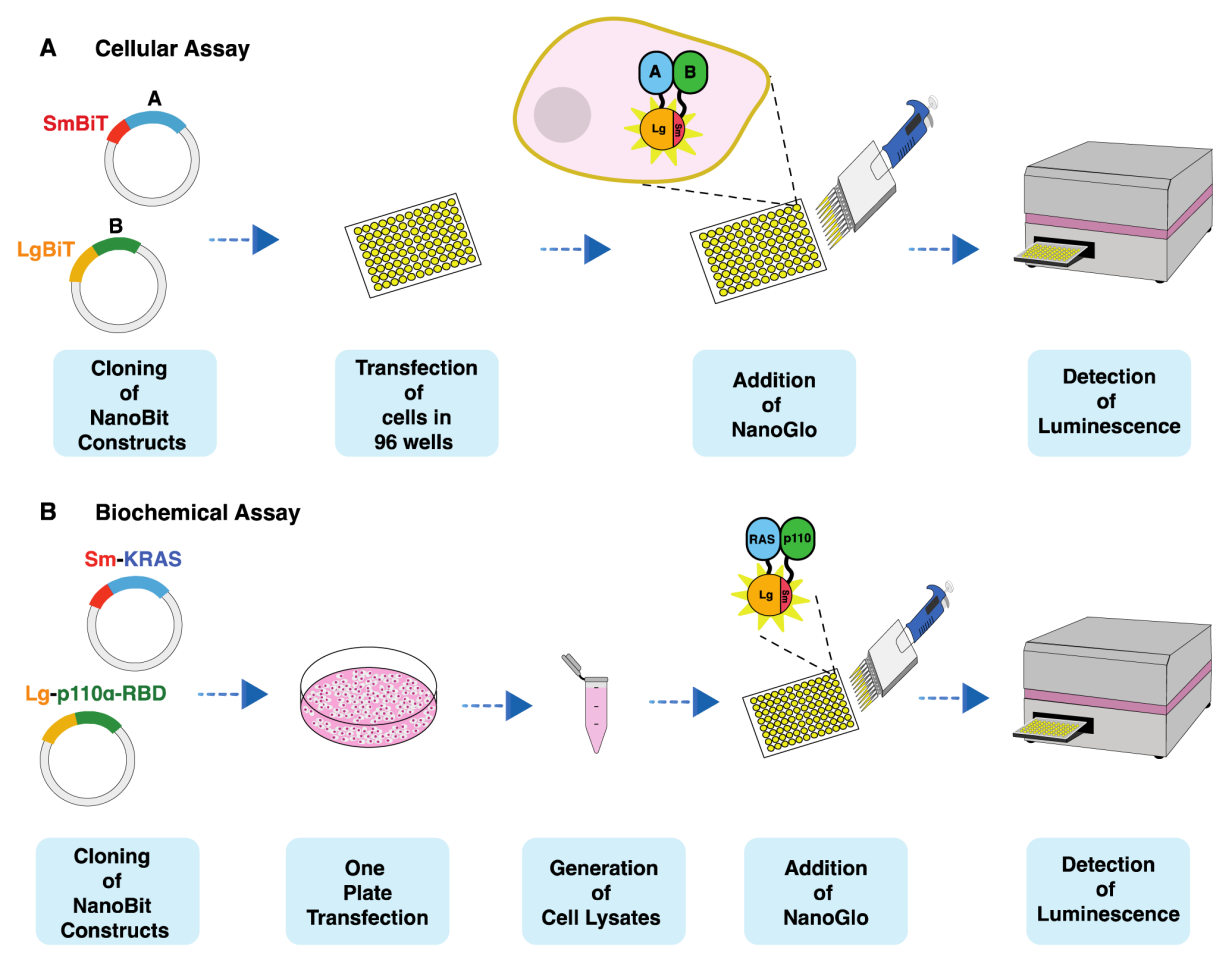
Schematic diagram of the steps involved in the NanoBiT biochemical assay (NBBA). The NanoBiT live cell assay, proteins of interest ( **A** and **B**) are cloned in the NanoBiT vectors expressing either the Sm (Small-BiT) or the Lg (Large-BiT). The constructs are transfected in individual 96 well plate, followed by the addition of 25 µl 1X Nano-Glo substrate for detecting the PPI ( **A**). In contrast, in the NBBA ( **B**), only one transfection is required, followed by cell lysis, quantification and titration of cell lysate into 384 well plates followed by the addition of 2 or 4 µl of 1X Nano-Glo for 10- or 20-µl reaction volume respectively for detecting the PPI.

The NanoBit ^®^ assay provides a tool for detecting protein-protein interactions in live cells ( Figure 1A). The assay is based on splitting the engineered luminescent protein NanoLuc ^®^ into two separate subunits, the small BiT (SmBiT, 1.3 kDa in size) and the Large BiT (LgBiT, 18 kDa in size). These SmBiT and LgBiT subunits (hereafter Sm and Lg) interact very weakly with an affinity of K _d_ = 190 µM, so their assembly to form an active luminescent complex only occurs upon the interaction of the separate binding partner proteins to which they are fused. The NanoBit cellular assay has proved successful in several cellular PPI studies ^16^. Nevertheless, cellular assays are usually more demanding, do not work for compounds that are not cell membrane permeable and do not produce the high throughput and consistency offered by biochemical assays ^17, 18^. Here we present the successful translation of the NanoBiT cellular assay to a biochemical assay, which we have termed the NanoBit Biochemical Assay (NBBA). The NBBA proves efficient in detecting both the strong and weak interactions between RAS/RAF and RAS/PI3K respectively. Furthermore, the NBBA is responsive to various types of RAS inhibitors and can be used in small and large scale screening.

## Results

Raw values for each experiment are available as *Underlying data* ^19^.

### HTRF assay is suitable for detecting the RAS/RAF interaction but not RAS/PI3K

**Figure 2.**
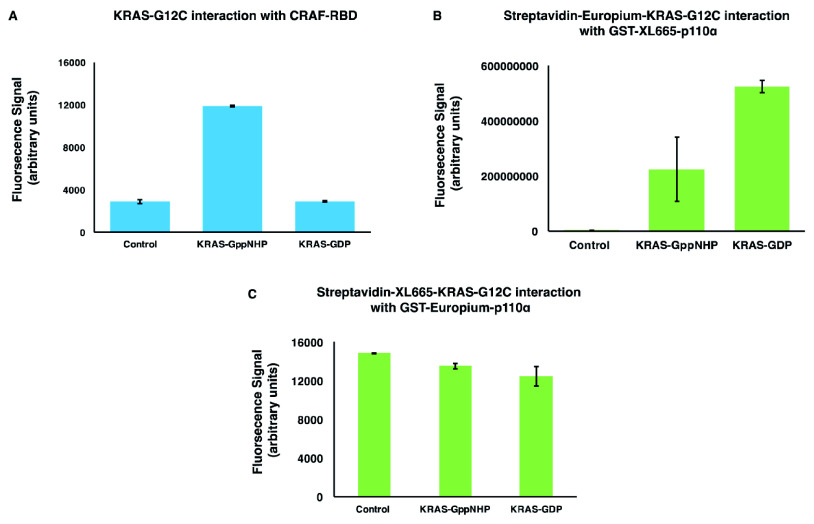
The homogenous time-resolved fluorescence (HTRF) assay is suitable for detecting the interaction of KRAS/CRAF but not KRAS/p110α. All data are produced from replicates (n=4). ( **A**) 5 nM Avi-KRAS was loaded with either GppNHP (GTP analogue) or GDP was labelled with streptavidin-Europium (donor beads), and mixed with 10 nM labelled GST-CRAF-RBD with anti-GST XL665 (acceptor beads). Control, contains are the donor and acceptor beads with TB (titration buffer). There is a clear signal of CRAF-RBD with KRAS_GppNHP but not with KRAS_GDP. ( **B**) 3 µM Streptavidin-Europium-KRAS_GppNHP or KRAS_GDP with 10 nM anti-GST XL665-p110α. The signal of fluorescence was too high due to the high concentration of Europium used in the experiments. ( **C**) 10 nM GST-Europium-p110α mixed with Streptavidin-XL665- KRAS_GppNHP or KRAS_GDP. The signal is lower than the experiment in ( **C**); however, the difference between the control (TB buffer or KRAS-GDP) and the positive interaction is very narrow, which makes it unsuitable for drug screening.

We chose homogeneous time-resolved fluorescence (HTRF) as an initial biochemical screening assay since it has previously been shown to be a suitable format for the measurement of active KRAS/CRAF-RBD binding interactions ^20^. An activated form of KRAS bearing the mutation G12C and the CRAF-RBD were expressed and purified from *Escherichia coli* and the KRAS loaded with GppNHP (a non-hydrolysable GTP analogue). With KRAS at 5nM and CRAF-RBD at 10 nM we obtained a clear signal of interaction between active KRAS-G12C-GppNHP and CRAF-RBD as compared to the inactive KRAS-G12C-GDP ( Figure 2A) ^19^. 

To determine if a similar specific response could be seen with p110α we used the full-length p110α fused to GST produced in baculovirus, as the isolated PI3K and their RBDs are known to be poorly soluble ^21^. Since p110α is not as soluble as KRAS, we kept the concentration of p110α low (10 nM) and added the KRAS at 3 μM (approximately the interaction K _d_). This posed a challenge for HTRF as the labelling reagents also needed to be at a high concentration, using large amounts of reagent and resulting in high background signals. KRAS was labelled with either streptavidin-europium or streptavidin-XL665; coupled with either p110α labelled with Anti-GST XL665 or streptavidin-europium, respectively. Only a low signal was achieved ( Figure 2B, C) ^19^ with KRAS-GppNHP compared to KRAS-GDP, suggesting that this approach was not promising for further follow up. Together, these results show that whilst the HTRF assay is appropriate for strong PPI, such as RAS/RAF binding, it is unsuitable for much weaker interactions such as RAS/p110α.

### Translation of the NanoBiT cellular assay to a biochemical assay

To pursue the development of an assay for relatively weak protein interactions, we conducted several experiments aimed at transforming the NanoBit cellular assay into a biochemical assay using cell lysate from mammalian HEK293 cells. In principle, the assay involves transfecting cells with SmBiT and LgBiT expression constructs containing the target proteins of interest, followed by harvest, lysis, lysate quantification, aliquoting and addition of the Nano-Glo ^®^ Live Cell Substrate (hereafter Nano-Glo) for detection ( Figure 1B). The pros and cons of using cell lysates versus live cells in PPI screens are outlined in Table 1.

**Table 1.** The advantages and disadvantages of the NanoBiT cellular assay vs the NanoBIT biochemical assay.

Live cell monolayer	Cell lysate (biochemical)
Working with monolayer cells is costly – in particular transfection reagents, Nano-Glo reagent and cell culture plates.	Relatively cost effective as large amounts of transfection reagent and Nano-Glo reagent are not required.
Technically challenging for high throughput screening – needs to be compatible with work under a sterile environment.	Suitable for high throughput screening and does not necessitate extensive work under sterile environment.
Time consuming – takes up to two days to seed and transfect cells.	Time efficient - lysate can be generated in bulk, aliquoted and stored in -80 for multiple usages.
Transfection efficiency may vary among wells, giving inconsistent results.	Transfection is only performed once, so all wells contain a uniform lysate.
Limited control over protein expression levels.	Protein concentration can be optimized by diluting the cell lysate in lysis buffer.
Proteins will be expressed in their natural environment, and will have undergone all post translational modifications.	
Positive hits obtained from the monolayer cells will indicate that they are also cell penetrable.	The positive hits from the cell lysate will give no indication if they are cell penetrable

We first tested the interaction of the KRAS-G12C (hereafter KRAS) oncogenic mutant with CRAF protein (hereafter RAF), to determine if the NanoBit assay was suitable for biochemical analysis. Both KRAS and RAF were cloned into the NanoBiT vectors (BiBiT vectors system) with the orientations Lg-KRAS and Sm-RAF, respectively, and co-transfected into HEK293 cells. The Lg and the Sm tags were positioned at the N-terminal KRAS and RAF, as RAS localizes on the cellular membrane via its C-terminal region and to ensure appropriate activation of RAF C-terminal kinase domain. Due to the strong interaction of RAS with RAF, we prepared a serial dilution of the cell lysate from 0.002–5 µg/µl in 20 µl reaction volumes ^13^.

**Figure 3.**
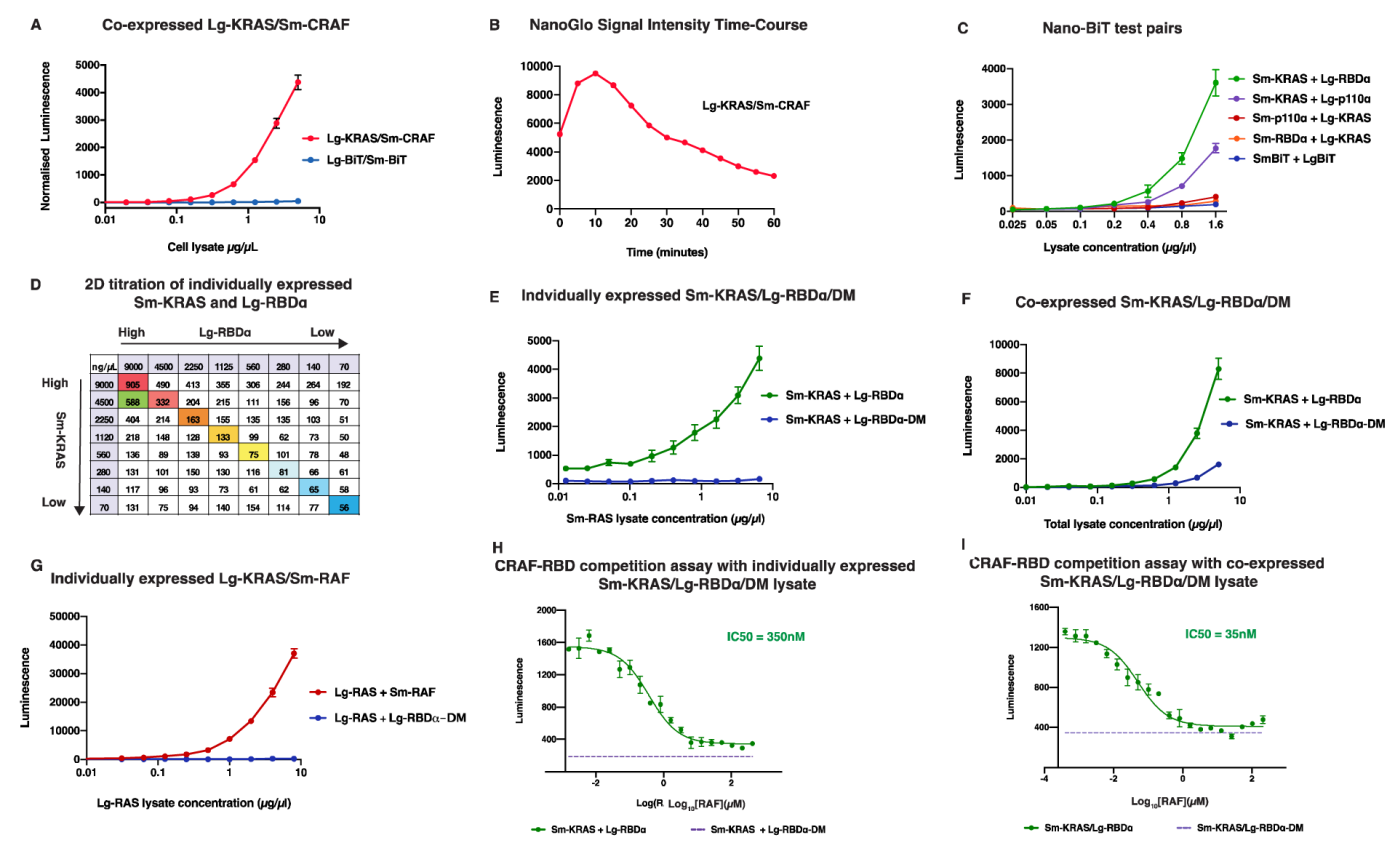
NanoBit biochemical assay (NBBA) expression, protein-protein interaction (PPI) detection and competitional assay. ( **A**) Titration of HEK293 cell lysate co-expressing Lg-KRAS/Sm-CRAF (1–5000 ng/µl). The negative control is the lysate expressing empty vectors of Sm and Lg-BiTs. ( **B**) Time course of the half-life of the Nano-Glo substrate after adding it a fixed concentration of the Lg-KRAS/Sm-CRAF. ( **C**) Different construct arrangements of the Sm and the Lg with KRAS, p110α, and RBDα tested by co-transfection into HEK293 cells. The addition of the Lg at the N-terminal of p110α and RBDα, and the Sm to KRAS gives a strong and reliable signal of interaction. The negative control lysate comes from the transection of HEK293 cells with empty Sm and Lg vectors. ( **D**) 2D titration of individually expressed Lg-RBDα and Sm-KRAS (from 0.07–9 µg/µl each). The coloured boxes illustrate the reduction in luminescence signal from the lower cell lysate concentrations. ( **E**, **F**) The detection of the Sm-KRAS and the Lg-RBDα protein interaction produced from either individually expressed ( **E**) or co-expressed HEK293 cell lysate ( **F**). **E**, Lg-RBDα lysate was used at 8 µg/µl and the Sm-KRAS was titrated at different concentrations. It required 0.2 µg/µl Sm-KRAS + 8 µg/µl Lg-RBDα to obtain a luminescence signal of ~1000 versus, 1 µg/µl from the co-expressed lysate ( **F**). ( **G**) Titration of HEK293 cell lysate individually expressing Lg-KRAS, with fixed concentration of cell lysate 300 ng/µl expressing either Sm-CRAF (red) or Lg-KRAS/RBDα-DM as a negative control. ( **H**, **I**) Competition assay titrating purified CRAF-RBD (0–400 µM) individually expressed ( **D**) or co-expressed ( **E**) cell lysate of Sm-KRAS/Lg-RBDα. **D**, individually expressed lysate using Sm-KRAS at 5 µg/µl with Lg-RBDα/DM at (2.3 µg/µl), gave an IC50 of about 300 nM. **E**, co-expressed Sm-KRAS/Lg-RBDα 1 µg/µl concentration, that gave an IC50 of about 35nM. Calculated IC50 values were determined using Prism 8.

In addition, we used only 4 µl of 1X Nano-Glo rather than the 25 µl that is required for live cells. We detected a clear signal increase of the Lg-KRAS/Sm-RAF interaction starting from 156 ng/µl cell lysate concentration and a lack of signal from the Sm/Lg BiT alone ( Figure 3A) ^19^. We then proceeded to perform a time course of the Lg-KRAS/Sm-RAF cell lysate to determine the half-life of the Nano-Glo signal. We found the signal to increase during the first 5 min of reaction and to fall again after 20 min ( Figure 3B) ^19^.

### The Lg-BiT enhances the solubility of poorly soluble proteins

We next employed the same methodology, co-transfection of HEK293 cells with the two constructs using the same BiBiT construct orientation, to detect the interaction of Lg-KRAS with either Sm-p110α or Sm-p110α-RBD (hereafter RBDα) in the cell lysates. Since the interaction between KRAS and p110α is substantially weaker than the RAS/RAF interaction, we titrated the cell lysate from 0.01–5 µg/µl ^14^. In the initial test, we failed to detect a significant signal of interaction between Lg-KRAS and Sm-p110α or Sm-RBDα ( Figure 3C) ^19^ and surmised that this was due to the poor solubility of the p110α and RBDα ^21^. Since the Lg-BiT is the larger part of the NanoLuc protein and is properly folded, we hypothesized that switching the NanoBiT tags and adding the Lg-BiT on the N-terminal of p110α and the RBDα, could improve the solubility of the proteins. We found that when co-transfecting Sm-KRAS with either Lg-p110α or Lg-RBDα, we now detected a clear increase in signal of interaction between Sm-KRAS/Lg-p110α and Sm-KRAS/Lg-RBDα, with an ideal lysate concentration range of 0.025–1.6 µg/µl ( Figure 3C) ^19^. This suggests that fusion to the Lg-BiT can indeed improve the solubility of poorly soluble proteins. Since the signal obtained from the Sm-KRAS/Lg-RBDα was significantly stronger than the one obtained with the Sm-KRAS/Lg-p110α interaction, we decided to continue our study using the isolated RBDα rather than full-length p110α. In addition, we constructed an RBDα negative control (termed Lg-RBDα-DM) by inserting two mutations in the RBDα (T208D and K227A) that are known to block the interaction of p110α with RAS ^12^.

### Co-expression of Sm-KRAS/Lg-RBDα is more efficient than individual expression in detecting the complex interaction

We additionally tested the signal from Sm-KRAS/Lg-RBDα complex formation and the efficiency of the luminescence by expressing both proteins separately in HEK293 cells. We applied a two-dimensional (2D) titration of both Sm-KRAS and Lg-RBDα in 96 well plates to determine the concentration of each protein needed to give an ideal signal of PPI that could be used for drug screening. We found that using the Sm-RAS lysate at 4.5 µg/µl with Lg-RBDα lysate at 9 µg/µl gave a luminescence signal around 588 RLU (Relative Luminescence Signal) – intermediate between the highest (905 RLU) and the lowest (56 RLU) signal, which could be suitable for drug screening ( Figure 3D) ^19^. However, it appeared that greater quantities of cell lysates were needed from individually expressed Sm-RAS and Lg-RBDα than with the co-expressed lysate to obtain a comparable signal. This was further tested by titrating both co-expressed and individually expressed Sm-KRAS and Lg-RBDα/DM (Lg-RBDα or Lg-RBDα-DM). We observed that in the individually expressed lysates we needed around 0.2 µg/µl Sm-KRAS + 8 µg/µl Lg-RBDα to obtain an RLU signal of ~1000, whereas in the co-expressed lysate we only required 1 µg/µl to reach a comparable level. The Lg-RBD-DM showed no signal increase with the individually expressed lysate, and only a slight signal increase (compared with the Sm-KRAS and Lg-RBDα signal) at higher concentrations in the co-expressed lysate ( Figure 3E, F) ^19^. However, in the case of Lg-KRAS and Sm-RAF, we did not witness a significant difference between co-expressed and individually expressed lysates when Lg-KRAS was titrated in the presence of 0.3 µg/µl Sm-RAF ( Figure 3G) ^19^. The differences in signal strength between the individually and the co-expressed Sm-KRAS/Lg-RBDα experiments could be explained by the fact that in the co-expressed format the PPI happens prior to cell lysis and produces a relatively stable protein complex.

### The NBBA is responsive to the inhibition of protein interactions

Since the RAS/RAF interaction is about 150-fold stronger than RAS/p110α, we used the RAF-RBD as a competitive inhibitor of the Sm-KRAS/Lg-RBDα interaction in the NBBA. We chose cell lysate concentrations that produce a signal of around 3–4-fold higher than the negative control to avoid high concentrations that may overwhelm the sensitivity of the assay in detecting PPI inhibition. We were also interested to compare the RAF-RBD inhibitory effect on Sm-KRAS/p110α complex produced from either individually or co-expressed lysates. Firstly, with individually expressed protein lysates, Sm-KRAS was used at a concentration of 5 µg/µl with 2.3 µg/µl Lg-RBDα/DM. Second, co-transfected Sm-KRAS/Lg-RBDα-DM cell lysates were used at 0.5–1 µg/µl. We then titrated purified RAF-RBD from 400 µM–0.015 nM. In both experimental formats, we observed a constant decline of the signal with the increase of the CRAF-RBD ( Figure 3H, I) ^19^. However, the individually expressed lysate format produced an estimated half-maximal inhibitory concentration (IC50) of 350 nM versus the co-expressed lysate IC50 35 nM which is closer to the generally accepted RAS/RAF complex affinity. This supports the notion that the co-expressed Sm-KRAS/Lg-RBDα cell lysate is the preferable format for the study of these PPIs.

### The NBBA is suitable for testing different types of inhibitors

**Figure 4.**
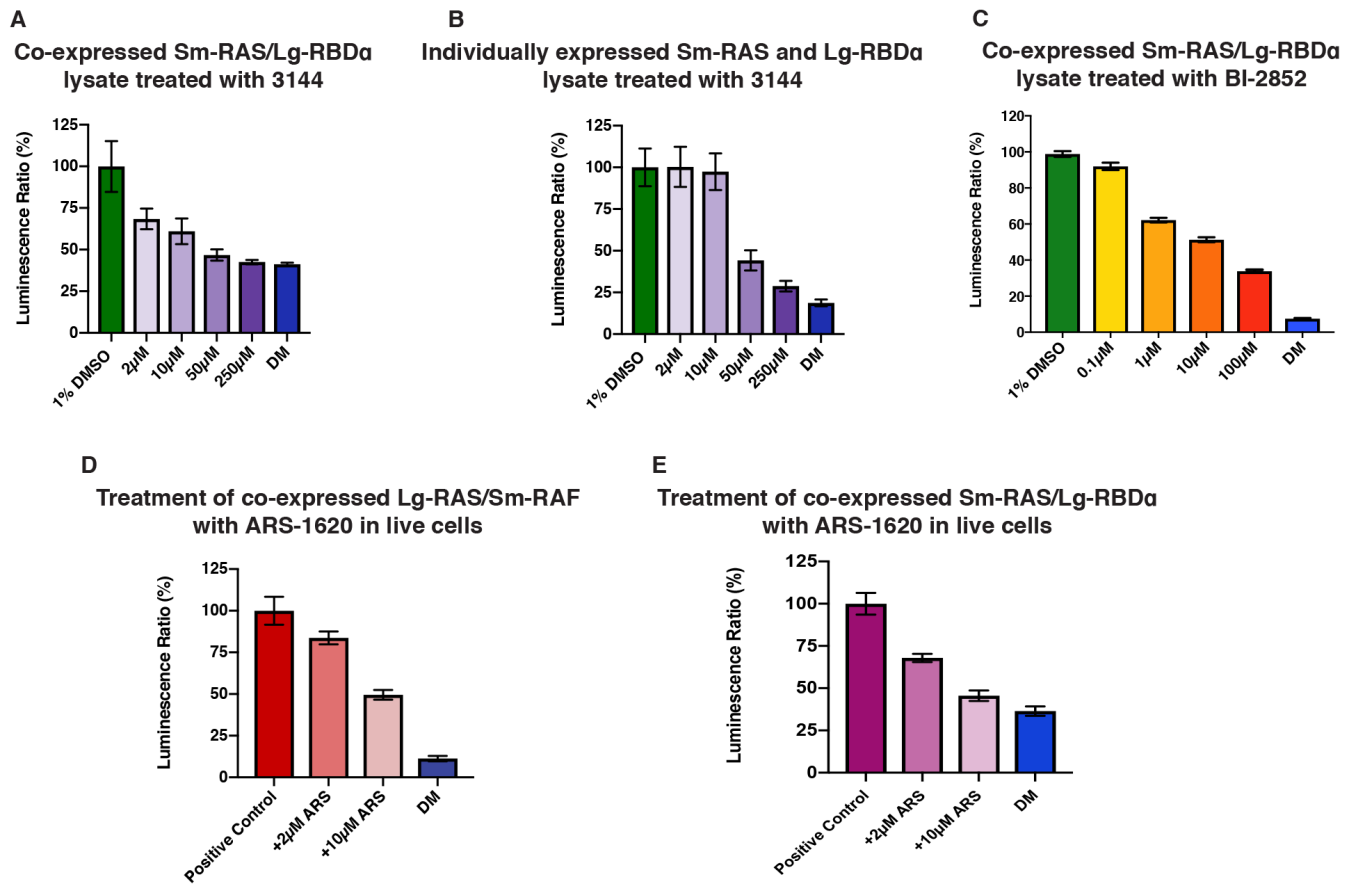
The NanoBit biochemical assay (NBBA) is responsive to different inhibitors. ( **A**, **B**) The treatment of co-expressed Sm-RAS/Lg-RBDα (1 µg/µl) ( **A**), or individually expressed Sm-RAS and Lg-RBDα at (0.5 µg/µl KRAS and 2.3 µg/µl Lg-RBDα) ( **B**), with several concentrations of the PAN RAS inhibitor 1344 (2, 10, 50 and 200 µM). The positive control was treated with 1% DMSO equivalent to the concentration of DMSO in the highest 1344 concentration (200 µM). ( **C**) Treatment of co-expressed Sm-RAS/Lg-RBDα using with the BI-2852 KRAS inhibitor at (0.1, 1, 10, and a 100µM) concentrations. ( **D**, **E**) Treatment of HEK293 cells expressing either, Lg-KRAS/Sm-RAF ( **D**) or Sm-KRAS/Lg-RBDα ( **E**) with two concentrations the KRAS-G12C specific covalent inhibitor ARS-1620 (2 and 10 µM).

We chose to investigate this system further by studying the efficiency of the NBBA response to other types of inhibitors. We selected three examples of recently described RAS inhibitors: Pan-RAS inhibitor 1344 (hereafter 1344), which is a weak binder of RAS (K _d_ ~ 17 µM), a stronger KRAS inhibitor BI-2852 (K _d_ ~ 750 nM), and mutation-specific covalent KRAS-G12C inhibitor, ARS-1620 ^22– 24^. In the case of 1344, we tested again the two types of cell lysate, co-expressed KRAS/Lg-RBDα/DM and individually expressed KRAS together with Lg-RBDα or Lg-RBDα-DM. The cell lysates were then treated with several concentrations of the 1344 inhibitor (2, 10, 50 and 250 µM) for 20 min prior to the addition of the Nano-Glo reagent. We found that 1344 was able to inhibit the Sm-KRAS/Lg-RBDα PPI in a dose dependent manner and did so more efficiently in the co-expressed lysate – similar to our earlier observations with RAF-RBD inhibition ( Figure 4A, B) ^19^.

Based upon the above observations, we decided to continue our studies using only the co-expressed lysate. We tested the effect of the KRAS inhibitor BI-2852 on the co-expressed Sm-KRAS/Lg-RBDα starting with lower inhibitor concentrations (0.1, 1, 10 and 100 µM) since it is a stronger inhibitor than 1344. The BI-2852 was also able to inhibit the Sm-KRAS/Lg-RBDα PPI in a dose dependent manner starting from around 1 µM concentration ( Figure 4C) ^19^.

Furthermore, we also tested the covalent RAS-G12C inhibitor ARS-1620 on the co-expressed Sm-KRAS/Lg-RBDα and Lg-KRAS/Sm-RAF PPI. The ARS-1620 predominantly targets the RAS-GDP state, therefore it is important to test its effects in live cells where the impact on GDP/GTP exchange can be assessed. Both Sm-KRAS/Lg-RBDα and Lg-KRAS/Sm-RAF were co-transfected in HEK293 cells for 48 hr. The cells were then treated with either 2 or 10 µM ARS-1620 for a further 4 hr. Cells were then harvested, lysed, sonicated and the cell lysis was quantified for PPI detection. We found that the ARS-1620 was indeed capable of reducing the signal of KRAS/Lg-RBDα and Lg-KRAS/Sm-RAF PPI at 2 µM and at 10 µM concentration ( Figure 4D, E) ^19^. These results demonstrate that the NBBA is also suitable for studying the effects of covalent inhibitors on PPIs.

### The NBBA can detect other PI3K family RBD interactions with RAS and has an excellent Z’ value

**Figure 5.**
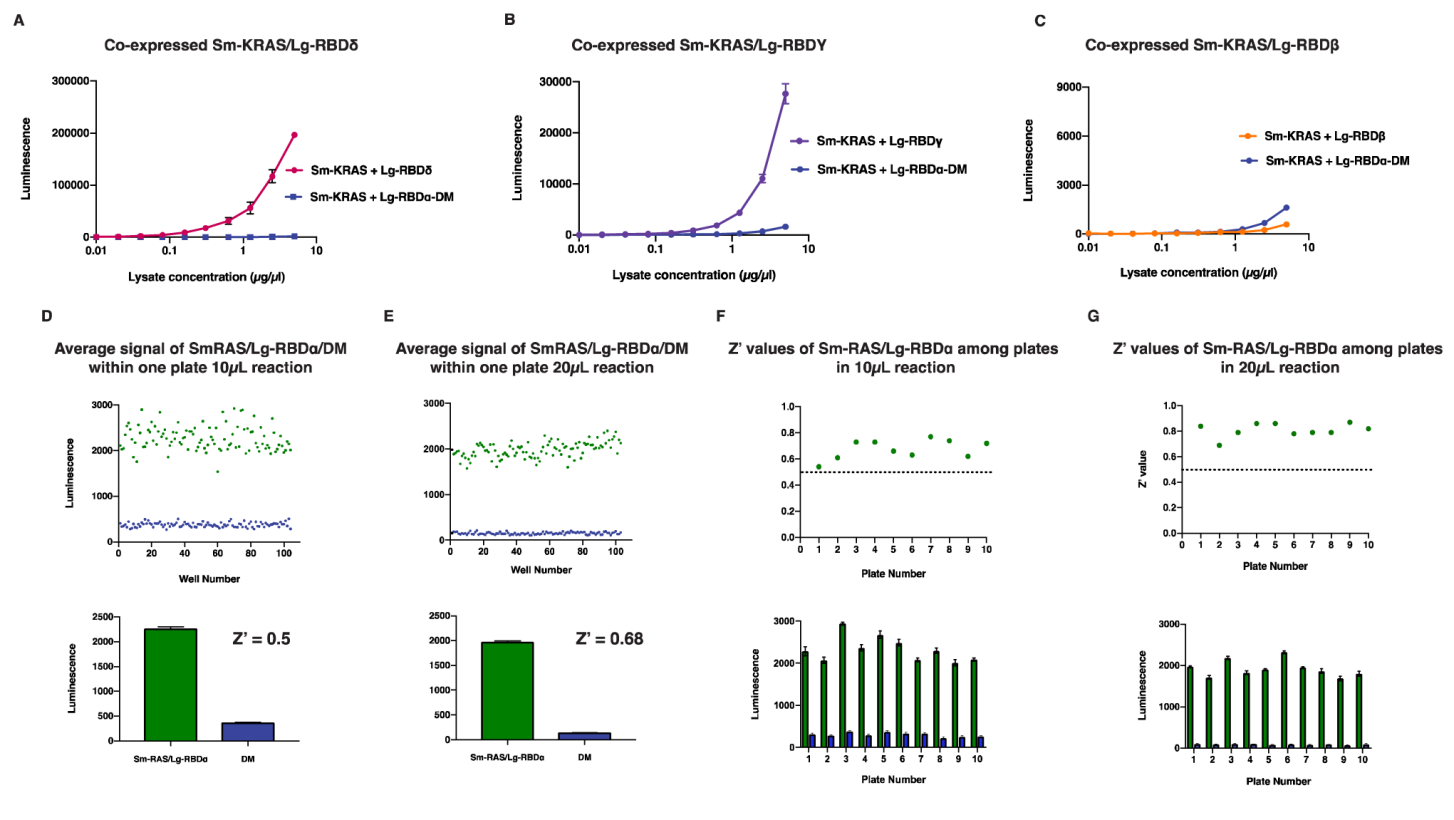
The NanoBit biochemical assay (NBBA) can detect other PI3K family RBD interactions with RAS and has an excellent Z’ value. ( **A**– **C**) Titration co-expressed Sm-KRAS with Lg-RBDδ ( **A**), RBDγ ( **B**) and RBDβ ( **C**). Both Lg-RBDδ and γ showed interaction with Sm-KRAS whereas Lg-RBD β did not. ( **D**, **E**) Co-expressed Sm-KRAS/Lg-RBDα titrated (at 1 µg/µl) in different wells (n=104) across a 384 well plate in 10 µl ( **D**) and 20 µl ( **E**) reaction volumes – along with the negative control Sm-KRAS/Lg-RBDα-DM. Top, scatter plots of luminescence signal arising from each well. Bottom, average of all 104 wells from the 10-µl (Z’ = 0.5) and 20-µl (Z’ = 0.68) reaction volume. ( **F**, **G**) Co-expressed Sm-KRAS/Lg-RBDα titrated (at 1 µg/µl) in several wells (n=6) across 10 plates in a 10 µl ( **G**) and 20 µl (H) volume – along with the negative control Sm-KRAS/Lg-RBDα-DM. Top, scatter plots of the average Z’ value from each plate. Bottom, average luminescence signal from each plate.

It was previously published that RAS proteins only interact with certain isoforms of PI3K, namely p110α, γ and δ, but not with p110β ^14^. We therefore decided to test whether the NBBA is capable of distinguishing the different interacting isoforms of PI3K with RAS. The RBDs of p110γ, δ, and β were expressed as fusions with the Lg-BiT and tested in co-transfection with Sm-KRAS. The cell lysates of Sm-KRAS with either Lg-RBDγ, δ or β, were titrated at various concentrations by serial dilution, Nano-Glo was added and the effects analysed. The results clearly showed that Sm-KRAS interacts with p110γ and δ, but not with p110β ( Figure 5A–C) ^19^.

In order to test if the NBBA would be suitable and sufficiently reproducible for measuring PPI in a drug screening setting, it was important to determine the Z’ factor value across different wells in a 384-well plate and across several different plates ^25^. The co-expressed Sm-KRAS/Lg-RBDα/DM lysate was aliquoted across multiple 384 well plates at 10 µl and 20 µl reaction volume – followed by the addition of Nano-Glo (2 and 4 µl, respectively). Both reaction volumes, 10 µl and 20 µl, showed excellent Z’ values of 0.5 and 0.7, respectively across the 384 wells, and Z’ value of above 0.5 across different plates, demonstrating that the NBBA is a robust and reliable assay for high throughput PPI and drug screening ( Figure 5D–G) ^19^.

### Establishing stable cell lines expressing the Lg-RAS/Sm-RAF for high-throughput screening

**Figure 6.**
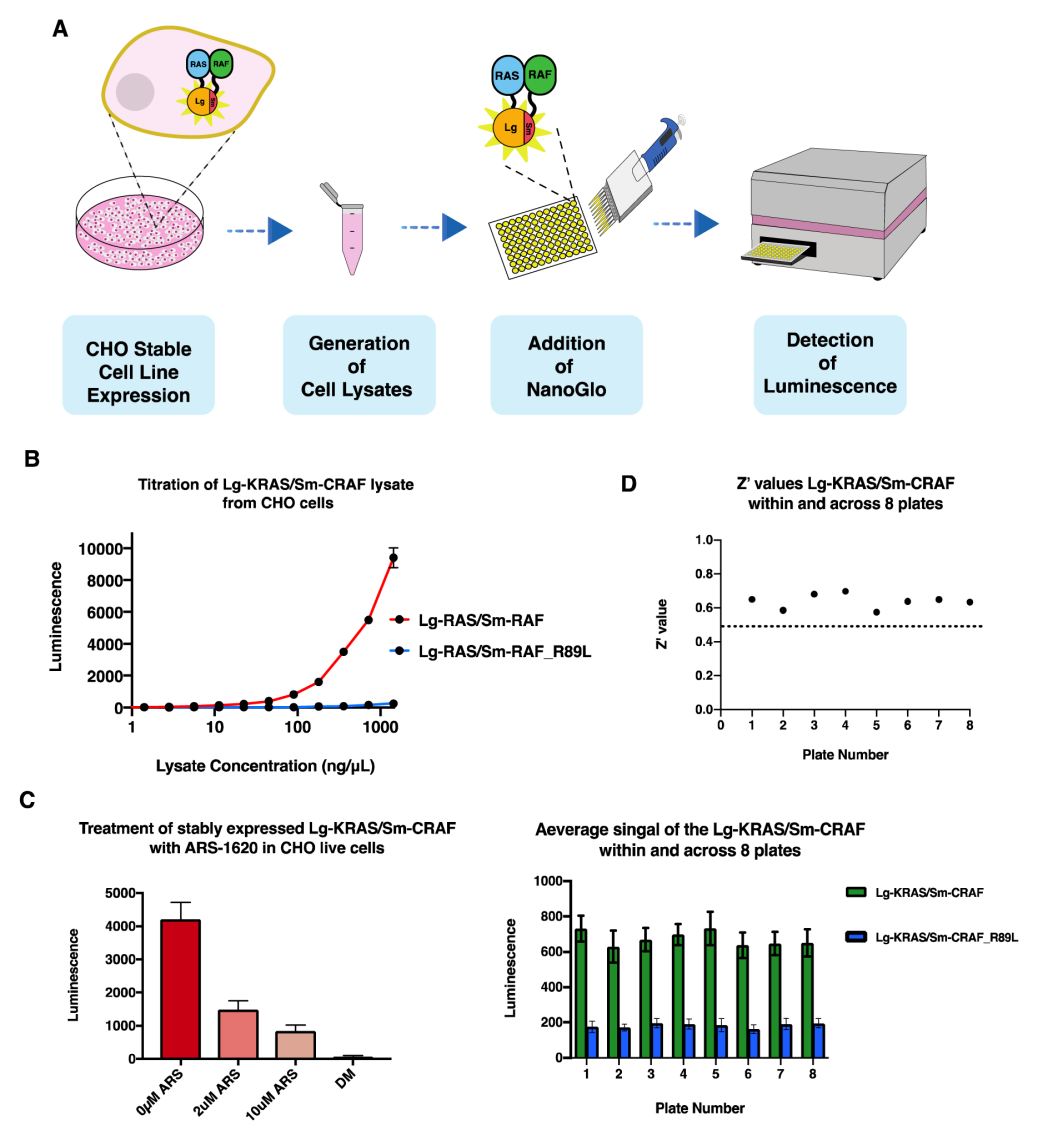
The NanoBit biochemical assay (NBBA) functions in stable cell line expression. ( **A**) Schematic diagram representing the steps required for the protein expression in stable cell lines. CHO stably expressing Sm-KRAS/Lg-CRAF cells are seeded on plates, cells are then harvested after 24hrs, titrated into 384 well plates followed by the addition of the Nano-Glo for detecting the protein interaction. ( **B**) Titration of CHO cell lysate stably expressing Sm-KRAS/Lg-CRAF. The signal of interaction is similar to the ones obtained from transient expression. ( **C**) Treatment of the Sm-KRAS/Lg-CRAF cell lysate arriving from CHO cells with ARS-1620. There is a clear inhibition of the KRAS/CRAF interaction at 2 µM and 10µM ARS-1620. ( **D**) Measuring the Z’ value, the Sm-KRAS/Lg-CRAF cell lysate in 8x 384 well plates using an automated dispenser. Top, scatter plot representing the Z’ value of each plate. Bottom, average luminescence signal from all 385 wells in each plate (green bars). Blue bars show the negative control (Sm-KRAS/Lg-CRAF-R89L).

We wanted to test the feasibility of generating cell lines that stably express the target proteins instead of employing transient expression. This would enable the production of cell lysate expressing the target proteins in a more robust and cost-effective manner. Thus, we generated CHO (Chinese hamster ovary) stable cell lines expressing the Lg-KRAS/Sm-CRAF protein complex (using the same vectors that were used in the transient transfection). The cells were seeded on plates (10 cm) and harvested after 24 hr, cell lysate was then quantified and titrated in 384-well plates as a 10 µl reaction volume ( Figure 6A) ^19^. We then applied our established experimental procedure to the cell lysate and observed that we can indeed obtain a signal of Lg-KRAS and Sm-CRAF similar to the transient expression signal ( Figure 6B) ^19^. In addition, as a negative control, we used a Sm-CRAF-R89L (hereafter Sm-RAS-M) construct that carries a single amino acid point mutation and is known to block interaction with ^26^. The lysate was responsive to ARS-1620 inhibitor ( Figure 6C) ^19^. Furthermore, we used an automated dispenser to titrate the cell lysate in eight different 384 plates to calculate the Z’ value. We found that all eight plates gave excellent Z’ values above 0.58 ( Figure 6D) ^19^, further supporting the notion that the NBBA assay is suitable for automated high throughput screening.

## Discussion

From these results, we conclude that the NBBA represents a useful development of the NanoBit live cell assay. The NBBA is simple and easy to use: for example, the cell lysates of the proteins of interest can be expressed, either by transient transfection or from stable cell lines, in high quantities, quantified and stored in aliquots at -80°C for multiple usages. Moreover, since the reaction volumes are only 10 or 20 µl, only small quantities of Nano-Glo reagent are needed, which makes it a cost-effective assay. The NBBA is a sensitive assay for detecting both strong and weak PPI. In addition, the Lg-BiT provides an extra function along with its essential luminescence role, as it can improve the solubility of poorly soluble proteins – such as the RBDs of PI3K. Furthermore, we showed that the NBBA is suitable for detecting PPI inhibition using four examples of inhibitors: a strong protein competitor protein RAF-RBD, a weak small molecule pan RAS inhibitor 1344, a stronger KRAS inhibitor BI-2852, and a covalent small molecule KRAS G12C inhibitor ARS-1620. In addition, the NBBA is suitable for use at small scale by transient expression in cells or at high scale using stable cell line expression of the target proteins of interest. Finally, the NBBA produces excellent Z’ values, which makes it a suitable assay for PPI or drug screening.

Interaction of RAS proteins with their effector enzymes underpins key aspects of both normal cellular growth control and also the aberrant proliferation seen in the roughly 15% of cancers that express mutationally activated RAS oncogenes. Many approaches have been taken to attempting to inhibit RAS oncoprotein function, but it has proven to be an extremely challenging drug target ^6^. One possible angle is to screen for small molecule inhibitors of the interaction of RAS with its effectors, either RAF or PI3K isoforms. Screening the RAS/RAF interaction has long been feasible, for example using HTRF assays or alpha screens. However, possibly due to the tight nature of this interaction, it has not proven possible to find inhibitors from screening libraries in this way. As the interaction of RAS with PI3K is some 150-fold weaker, we surmise that it may be far easier to detect compounds in screens that will interfere with this weak interaction. We can speculate as to why the NBBA overcomes previous difficulties in developing reliable high throughput assays for this interaction of RAS with PI3K: one possibility is that the interaction between these two proteins is stabilized to some extent by the very weak (~200 µM) interaction of the fusion split luciferase partners ^14^. In addition, the correct post-translational modification of the proteins achieved in this human cell line derived expression system might also prove advantageous. In any case, the NBBA for the interaction of RAS with PI3K may provide an ideal setting for identifying inhibitors of this interaction in high throughput screens. More broadly, this biochemical, cell-free derivative of the NanoBiT split-luciferase cell-based assay may be very useful for high throughput screening for inhibitors of a number of important protein-protein interactions.

## Methods

### Construct generation and cloning

**Figure 7.**
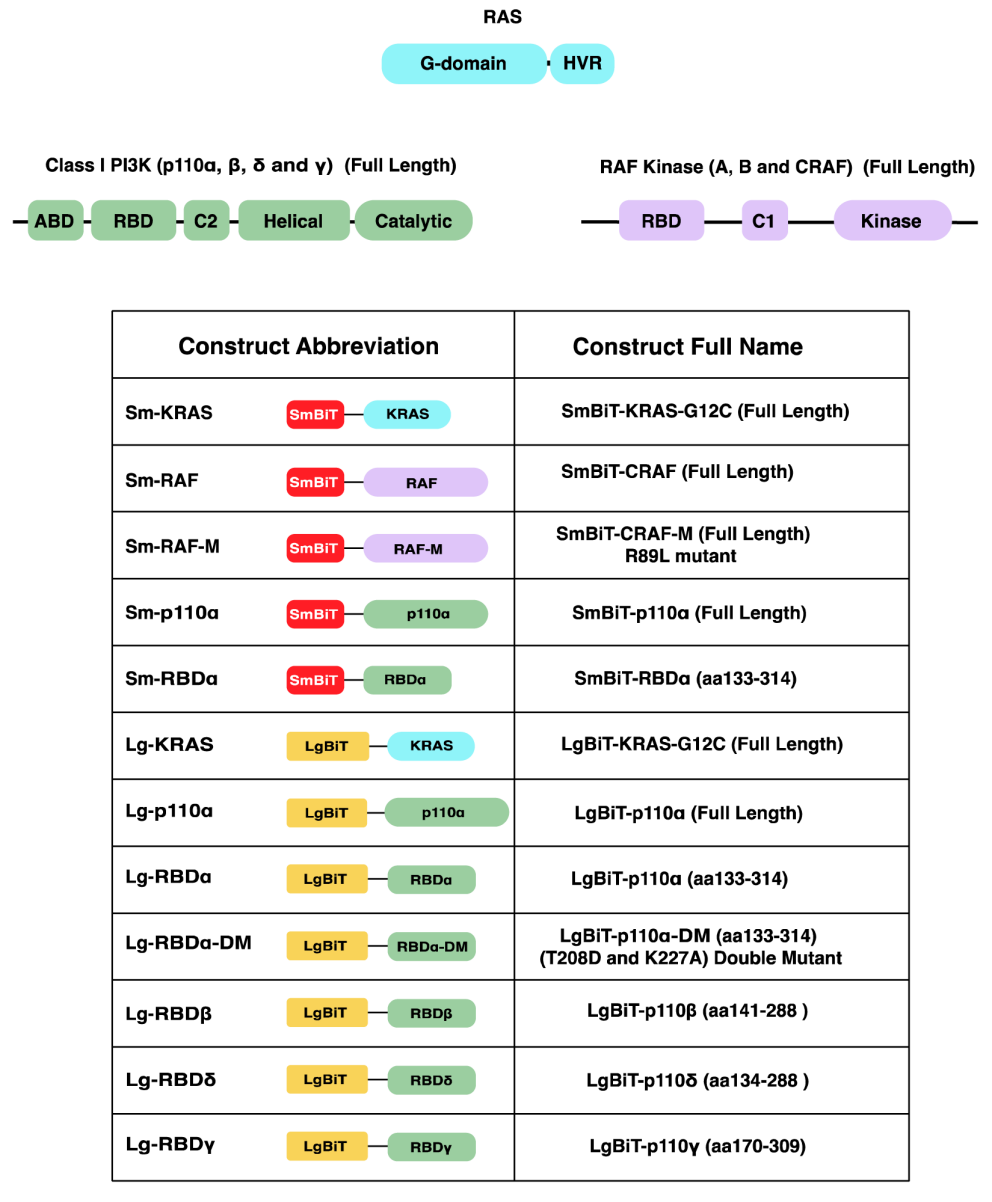
Abbreviations of SmBiT- and LgBiT- tagged protein constructs used in the NanoBiT assay. A diagram (top) to represent the domain structures of the current proteins used in the NanoBiT assay. The table illustrates the shorthand name and the full-length name of the SmBiT and LgBiT constructs used, with illustrations that correspond to the domain structure diagrams above.

All constructs with their abbreviations are listed in Figure 7. All inserts of the constructs used in this study are derived from human cDNA. The following full-length cDNA KRAS-G12C and p110α, and the PI3K-RBDs (RBDα aa133-314, RBDβ aa141-288, RBDδ aa134-288 and RBDγ aa170-309) were cloned using BiBiT NanoBiT vectors: pBiT2.3-N [CMV/SmBiT/Blast] Vector (pRSG199) or pBiT1.3-N [CMV/LgBiT/Hyg] Vector (pRSG197), using InFusion ^®^ Cloning and primer design (Takara Bio) using SacI and BamHI restriction sties. A list of the primer sequences used in this study is in Table 2. Both full-length Lg-KRAS-G12C and Sm-RAF were provided by Promega ready cloned into pBiT1.1-N [TK/LgBiT] and pBiT2.1-N [TK/SmBiT], respectively. Both Lg-KRAS-G12C and Sm-RAF were also sub-cloned in BiBiT NanoBiT vectors. The p110α regulatory subunit (p85α) was sub-cloned into pcDNA3 vector.

**Table 2.** List of primer sequences that have been used in this study.

Forward KRAS Full length – LgBiT/ SmBiT SacI	CGAGCGGTGGAGCTCAGATGACTGAATATAAACTTGTGG
Reverse KRAS Full length – LgBiT/ SmBiT BamHI	AGAACCGCCGGATCCCTACATAATTACACACTTTGTCTTTGAC
Forward p110α full length LgBiT/ SmBiT SacI	CGAGCGGTGGAGCTCAGATGCCTCCAAGACCATCATCAGG
Reverse p110α full length LgBiT/ SmBiT BamHI	AGAACCGCCGGATCCCTAGTTCAATGCATGCTGTTTAATTGTG
Forward RBDα 133-314 LgBiT/SmBiT SacI	CGAGCGGTGGAGCTCAGGATCCAGAAGTACAGGACTTCCG
Reverse RBDα 133-314 LgBiT/SmBiT BamHI	AGAACCGCCGGATCCCTAAGCTGTGGAAATGCGTCTGG
Forward RBDβ 134-288 LgBiT SacI	CGAGCGGTGGAGCTCAGAAGGATCCTGAAGTAAATGAATTTCG
Reverse RBDβ 134-288 LgBiT BamHI	AGAACCGCCGGATCCCTACTTGCAGCATTCCACAAGTATAAAATGG
Forward RBDδ 134-288 LgBiT SacI	CGAGCGGTGGAGCTCAGTTGTGCGACCCAGAAGTGAACG
Reverse RBDδ 134-288 LgBiT BamHI	AGAACCGCCGGATCCCTACCTCGCCATGCGGGATGAG
Forward RBDλ 170-309 LgBiT SacI	CGAGCGGTGGAGCTCAGGACGATGAGCTGGAGTTCACG
Reverse RBDλ 170-309 LgBiT BamHI	AGAACCGCCGGATCCCTACGTGTCCAGTACCACGTGAATC
Forward p110α T208D LgBiT	AATGACAAGCAGAAGTATGATCTGAAAATCAACCATGAC
Reverse p110α T208D LgBiT	GTCATGGTTGATTTTCAGATCATACTTCTGCTTGTCATT
Forward p110α K227A LgBiT	AAGCAATCAGGGCAAAAACTCGAAG
Reverse p110α K227A LgBiT	CAACATACTTCGAGTTTTTGCCCTGATTGCTTCAGCAAT
Forward CRAF K89L LgBiT	AAAGCACTCAAGGTGTTGGGCCTGCAACCAGAG
Reverse CRAF K89L LgBiT	CTCTGGTTGCAGGCCCAACACCTTGAGTGCTTT

PCR amplification of full length or target fragments of cDNA were carried out using the reagents and conditions listed in Table 3. Following agarose gel electrophoresis of PCR products, DNA was extracted and purified with the QIAquick PCR Purification Kit according to the manufacturer’s instructions. Inserts and vectors were added in an approximate molar ratio of 1 (vector): 8 (insert) and left at room temperature for 1 hours, followed by transformation. All new vector/insert mix were transformed into *E. coli* DH5-α competent cells: 20 min incubation on ice, 45 s heat shock at 42°C, followed by 3 min incubation on ice, then grown in SOC (Super Optimal Broth) medium for 1 hour prior to distribution onto Kanamycin LB agar plates prior to growth overnight (o/n). Colonies were picked and inoculated in LB broth o/n with kanamycin (60 μg/ml). Minipreps and Sanger sequencing of plasmids were carried out by the Genomics Equipment Park at the Francis Crick Institute. Minipreps were re-transformed and inoculated in 250 ml LB broth overnight and maxipreps were carried out using the QIAGENⓇ Plasmid Maxi Kit according to the manufacturer’s instructions. Maxiprep plasmids were used for the transfection in HEK293 cells. For mutant constructs, site-directed mutagenesis (SDM) of previously cloned SmBiT (RAF) and LgBiT (p110 RBDα) constructs was performed with PCR and plasmids were digested with 1 μl Dpn1 restriction enzyme (10 U/μl) (NEB) at 37°C for 1 hour – followed by transformation in DH5-α as above.

**Table 3.** PCR reagents and conditions. ( **A**) Table of reagents (KOD polymerase, Novagen, C133158, primers Sigma Life Science) and volumes used for the PCR amplification of inserts and vectors which were used to clone new SmBiT and LgBiT constructs. All reactions were carried out at a total volume of 50 μl. ( **B**) The conditions used for PCR amplification for cloning and SDM.

Reagent	Volume µL
KOD Hot Start Polymerase Buffer 10X	1
MgSO _4_ 25mM	3
dNTPs 2mM	5
KOD Hot Start DNA Polymerase	1
Forward Primer 10µM	1.5
Reverse Primer 10µM	1.5
Deionised H _2_O	37
DNA template (20-25ng)	X
Total	50

**Table 3.** PCR reagents and conditions. ( **B**) The conditions used for PCR amplification for cloning and SDM.

Step	Temperature °C	Time	Cycle X
Denaturation	95	3 (min)	
Denaturation	95	30 (sec)	25X
Annealing	60	30 (sec)	
Extension	72	3 (min)	
Extension	72	10 (min)	
Incubation	12		

### Transfection and lysis of HEK293 cells

HEK293 cells were acquired as growing cultures from the Francis Crick Cell Services Platform and maintained (at 37°C and 5% CO _2_) in DMEM (Dulbecco's Modified Eagle Medium) (Gibco ^TM^, 41966-029) supplemented with fetal bovine serum (FBS) (v/v 10%) (SIGMA F7524), L-Glutamine (3 mM) (SIGMA G7513), and Penicillin-Streptomycin antibiotics (100 units/ml) (SIGMA P4333). Prior to transfection, cells were seeded into 10-cm or 15-cm dishes for 24 hours. Co-transfection and individual transfection of SmBiT and LgBiT constructs were carried out according to the manufacturer’s instructions using FuGene HD Transfection Reagent (Promega, E2311) diluted with Opti-MEM ^TM^ (Gibco ^TM^, 51985-026) at a ratio of 3 (FuGene.): 1 µg (DNA). For individual transfection, 2 µg and 7 µg DNA were used in 10-cm and 15-cm dishes, respectively. In co-transfection experiments, 4 µg and 14 µg DNA in total were used for 10-cm and 15-cm dishes, respectively. In the case of Sm-KRAS and Lg-p110α co-transfection, we also transfected an equal amount of p85α-pcDNA, to ensure the stability and function of p110α protein.

After 48 hr of transfection, media was removed and the cells were washed once with ice cold phosphate-buffered saline (PBS) buffer. The cells were then harvested in 1X Passive buffer (Promega), sonicated for 10 s and centrifuged at 4°C for 10 min. The cell lysate was then quantified using a Protein Assay Kit (Bio-RAD, 500-0114), aliquoted (30 µl in PCR tubes), snap frozen in liquid nitrogen and then stored at -80°C.

### NBBA protocol for PPI detection

Cell lysates after quantification were titrated by 2-fold serial dilution using titration buffer (TB; 25 mM Tris pH8, 100 mM NaCl, 0.5 mM TCEP) in CorningTM 384-well plates (Greiner 784904). The concentration of the titration range was based on the affinity of interaction between the target proteins (Lg-KRAS/Sm-RAF 0.002–5 µg/µl in 20 µl and Sm-KRAS/Lg-PI3K proteins, from 0.01–10 µg/µl). The Nano-Glo Live Cell Substrate (Promega, N2012) was diluted in (Nano-Glo Live Cell Substrate (LCS) buffer, Promega, N2068) 1:20 to obtain 1X concentration, and added as 4 µl/20 µl reaction or 2 µl/10 µl reaction. Plates were then centrifuged 1000 rpm for 10–15 seconds and the luminescence signal was recorded immediately at time zero and every 5 min for 45 min, using PheraStar FSX Detection System (BMG Labtech). Negative controls, either Sm-RAS/Lg-RBDα-DM or Lg-RAS/Sm-RAF-M, at the same cell lysate concentration as the target PPI of interest were run in all experiments. These negative controls produced an average signal from 200–300 RLU. A signal of positive PPI was considered if it was above 3–4-fold higher than the signal arising from the negative.

Typical cell lysate serial dilutions for NBBA were produced as follows:

For a 10µl reaction example: for a titration range 0.002–5 µg/µl (12 titrations), 8 µl of TB was added to all tubes (with the exception of the first tube). The cell lysate was diluted to 5 µg/µl in 16-µl volume. Next, 8 µl was taken from the first tube and mixed with the 2 ^nd^ tube, then 8 µl was taken from the 2 ^nd^ tube and mixed with the 3 ^rd^ tube. This process was repeated to the last titration concentration 0.002 µg/µl (tube 12). This was followed by the addition of 2µl 1X Nano-Glo to all 12 tubes.

**Figure 8.**
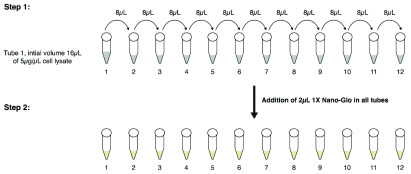
Schematic representation of a 10 µl NBBA reaction preparation. Step 1: An example of a cell lysate titration experiment ranging from 0.002–5 µg/µl concentration (12 tubes). The cell lysate will be diluted in TB at a concentration of 5 µg/µl in 16µl volume. The rest of the tubes will contain 8 µl TB. The titration steps start by taking 8µl from the 1 ^st^ tube and mixing it in the 2 ^nd^ tube, this step is repeated until tube 12. Step 2: The addition of 2 µl Nano-Glo reagent in all tubes. The above steps present the NBBA reaction preparation in tubes, however the same procedures are also applied in 384 well plates. For the 20 µl NBBA reaction, the same steps are the same with the exception that the first tube will contain 5 µg/µl cell lysate in 32 µl volume, followed by the addition of 4µl 1X Nano-Glo.

For a 20 µl reaction example: similar to above, with the exception that first the cell lysate was diluted at 5 µg/µl in 32-µl volume. Then, 16 µl from the first tube was used for mixing with the rest of the tubes (already containing 16 µl TB). This was followed by the addition of 4 µl of 1X Nano-Glo. A schematic presentation of the titration steps is illustrated in ( Figure 8).

Note 1: as protein expression will vary to some extent in each transfection, we ensured with every new lysate that we applied the above titration steps to determine the optimum lysate concentration needed to detect a reliable interaction signal (3–4 fold higher than the negative control). As described above, the cell lysate was then aliquoted, snap frozen in liquid nitrogen and stored in the -80°C.

Note 2: When cell lysate aliquots were thawed after storage at -80°C, we also repeated the above titration steps to ensure that the PPI was unaffected by freezing and had not lost activity.

### NBBA protocol for PPI inhibition

In all the PPI inhibition experiments we adopted the following procedure:

The cell lysate concentration of a positive PPI was selected at the point where it gave a luminescence signal 3–4-fold higher than the negative control (i.e. positive PPI signal 1000–1200 RLU and the negative control 200–300 RLU). This was to ensure that the lysate concentration was sufficiently high to detect the PPI, but not too high to detect a weak PPI inhibitor.

After incubation of the inhibitors with the cell lysate (producing the PPI of interest), and the addition of Nano-Glo, plates were centrifuged (as above) and the signal was collected immediately for 45 min.

### Competition assay

Co-expressed Sm-KRAS/Lg-RBDα 1 µg/µl, and the individually expressed Sm-KRAS, 0.5 µg/µl and Lg-RBDα 5 µg/µl were aliquoted in 19 wells. Lg-RBDα-DM served as a negative control. Purified RAF-RBD was titrated in all wells in a serial 2-fold dilution from 4–0.000015 µM. The plate was incubated on ice for 15 min, followed by the addition of 4 µl 1X Nano-Glo (per 20-µl reaction volume), and the luminescence signal was measured.

### 1344 Pan RAS inhibitor experiment

The co-expressed Sm-KRAS/Lg-RBDα/DM at 0.5 µg/µl, and the individually expressed Sm-KRAS with Lg-RBDα or Lg-RBDα-DM (0.5 µg/µl KRAS and 2.3 µg/µl Lg-RBDα/DM), were aliquoted in multiple wells, and the 1344 was added at (2, 10, 50 and 250 µM) and incubated on ice for 20 min. Following addition of 4 µl 1X Nano-Glo (per 20-µl reaction volume), the luminescence signal was measured. The highest concentration of the 1344 inhibitor (250 µM) contained a 1%DMSO in the final concentration of NBBA reaction, therefore we used a control reaction of 1%DMSO alone as a vehicle.

### ARS-1620 experiment

HEK293 cells were seeded in 10-cm plates, and co-transfected with Sm-RAS and Lg-RBDα/DM (as described above). After 48 hr of transfection, the cells were treated with 2 and 10 µM ARS-1620 for 4 hr. The cells were then harvested and lysed in Passive buffer, sonicated and the cell lysates were quantified. The ARS-1620 cell lysates were aliquoted in multiple wells along with the control (untreated) Sm-KRAS/Lg-RBDα cell lysate). Following addition of 4µl 1X Nano-Glo (per 20µl reaction volume), the luminescence signal was measured.

### Stable cell line development and maintenance

Lg-KRAS and Sm-CRAF were selected for the generation of stable cell lines. Constructs were transfected and selected separately. ExpiCHO-S cells were diluted to a density of 2×10 ^6^ cells/mL in 10 mL of Gibco ExpiCHO Expression Medium (Thermo Fisher Scientific). For transfection, 2 μg of DNA was diluted in reduced serum medium Opti-Pro (Thermo Fisher Scientific) to a total volume of 0.6 ml. Transfection reagents ExpiFectamine CHO (Thermo Fisher Scientific) were diluted at a ratio of 1:2.7 (plasmid: reagent) in reduced serum medium in a total volume of 0.6 mL. The solution was incubated at room temperature for 1 minute. After incubation, diluted DNA and transfection reagents were mixed and incubated for 5 minutes. The mixture was added to the cells drop by drop. Cells were incubated at 37°C in a 125-rpm shaker incubator with 8% CO _2_ for 24 hours and then diluted to 1×10 ^6^ cells/mL in 30 mL medium with 250 μg/mL of Geneticin (Thermo Fisher Scientific). Every 3–4 days, cells were selected and diluted in medium with Geneticin until they reached the usual ExpiCHO-S cell division rate of 3 times every 24 hours. Once the division rate became stable and cell viability was maintained at >98%, transfection of the second DNA constructs was carried out using the same procedure as the first transfection. Transfected cells were selected by both 250 μg/mL of Geneticin and 200 μg/mL of Hygromycin B (Biovision).

### Protein expression and purification

KRAS-G12C expression, purification and loading with GppNHP (Sigma G0635-5MG) or GDP (Sigma G7252) was performed using established protocols ^14^.

p110α: details outlining construction of plasmids used to express the p110α and p85α subunits of PI3K are given previously ^14^. Sf21 (Life Technologies) insect cells were co–infected with baculoviruses encoding GST-fusions of the p110α and p85α subunits and cultures allowed to grow at 27°C with shaking for three days. Cell pellets were then harvested and stored at -80°C until required. For purification, the thawed cell pellets were resuspended in buffer containing 50 mM HEPES (pH 8.0), 250 mM NaCl, 10% glycerol, 10 mM ß-glycerophosphate, 1 mM EDTA, 1 mM NaF, 10 mM benzamidine, 1 mM DTT and protease inhibitor cocktail (Roche), lysed by sonication and centrifuged to remove insoluble material. PI3K complexes were extracted from the soluble fraction of the lysate by affinity chromatography using glutathione agarose (GE Healthcare). Complexes were then cleaved from the resin by overnight digestion with HRV 3C protease, purified by gel-filtration and snap frozen in buffer containing 50 mM HEPES (pH8.0), 200 mM NaCl, 5% glycerol, 1 mM DTT.

### HTRF assays

KRAS-G12C/CRAF-RBD interaction: biotinylated RAS-G12C_GppNHP or GDP were labelled with the donor dye Streptavidin-Europium, and GST-CRAF-RBD was labelled with anti-GST XL665 dye as an acceptor. Anti-GST XL665-CRAF-RBD was mixed at 10nM with 5nM of either Streptavidin-Europium-RAS-G12C_GppNHP or GDP, incubated for 15min and data collected using the plate reader (PheraStar FSX Detection System (BMG Labtech)).

KRAS-G12C/p110α interaction: for the first experiment, 10 nM of Anti-GST-p110α labelled with XL665 (acceptor) were mixed with 3µM Streptavidin-Europium-RAS-G12C _GppNHP or GDP (donor). For the second experiment, 10nM of Europium-GST-p110α (donor) was mixed with Streptavidin- XL665-RAS-G12C _GppNHP or GDP (acceptor). In both experiments the reactions were incubated for 15min and data collected as above. 

### Data analysis

All experiments were performed in replicates of n=3, including the blank control wells (Nano-Glo in titration buffer). At the selected time points, measurements of all reactions were averaged and subtracted from the average signal of the blank control value. The average signals were plotted and fitted to lines and curves using GraphPad Prism 8. Z-prime (Z’) values, the statistical parameter used to assess the quality of a screening assay, were calculated using the formula:

$$Z'=1-\frac{3\sigma_{+}+3\sigma_{-}}{\left|\mu_{+}+\mu_{-}\right|}$$

where σ _−_ and σ _+_ represent the standard deviation of the luminescence signal of the positive control (Sm-KRAS and Lg-RBDα) and negative control (Sm-KRAS and Lg-RBDα-DM) respectively and μ _−_ and μ _+_ represent the mean luminescence signal of the positive control and negative control respectively. 0 < Z’ < 0.5 represents a ‘do-able assay’ and 0.5 ≤ Z’ < 1 represents an ‘excellent assay’ ^25^.

## Data availability

### Underlying data

Open Science Framework: NBBA (NanoBit Biochemical Assay). https://doi.org/10.17605/OSF.IO/MGKQV ^19^.

This project contains the following underlying data:

**•   All plates analysis (CSV):**
All the plates were analysed to obtain the mean and the SEM (Standard error mean) and plot the data for each experiment/figure.
**•   Figure 2–Figure 6 (CSV):**
Figure 2 A–C: HTRF assay of KRAS with RAF and p110α
Figure 2 A–C: HTRF assay of KRAS with RAF and p110α, analysis
Figure 3A: Titration of Lg-KRAS/Sm-RAF
Figure 3A: Titration of Lg-KRAS/Sm-RAF, analysis
Figure 3B: Nano-Glo Time course
Figure 3C: LgRAS_Sm-p110α expression
Figure 3C: Sm-KRAS/Lg-p110α and Lg-RBDα
Figure 3C: LgRAS_Smp110α expression, analysis
Figure 3D: 2D titration of Sm-KRAS/Lg-RBDα
Figure 3D: 2D titration of Sm-KRAS/Lg-RBDα, analysis
Figure 3E: Individual expression Sm-KRAS/Lg-RBDα
Figure 3E: Individual expression Sm-KRAS/Lg-RBDα, analysis
Figure 3F: Co-expression of Sm-KRAS/Lg-RBDα
Figure 3F: Co-expression of Sm-KRAS/Lg-RBDα, analysis
Figure 3G: Individually expression of Lg-KRAS/Sm-RAF
Figure 3G: Individually expression of Lg-KRAS/Sm-RAF, analysis
Figure 3H: Individually expressed Sm-KRAS/Lg-RBDα Competition experiment
Figure 3I: Co-expressed Sm-KRAS/Lg-RBDα Competition experiment
Figure 4A–B: PAN RAS inhibitor 1344
Figure 4A–B: PAN RAS inhibitor 1344, analysis
Figure 4C: KRAS inhibitor BI-2852 experiment
Figure 4C: KRAS inhibitor BI-2852 experiment, analysis
Figure 4D: ARS-1620 inhibitor with Lg-RAS/Sm-RAF
Figure 4D: ARS-1620 inhibitor with Lg-RAS/Sm-RAF, analysis
Figure 4E: ARS-1620 inhibitor with Sm-RAS/Lg-p110α
Figure 4E: ARS-1620 inhibitor with Sm-RAS/Lg-p110α, analysis
Figure 5A–C: Co-expression of Sm-KRAS/Lg-RBDα delta, gamma and beta
Figure 5A–C: Co-expression of Sm-KRAS/Lg-RBDα delta, gamma and beta, analysis
Figure 5D: Z' of Sm-KRAS/Lg-RBDα 10µL reaction across 1 plate
Figure 5D: Z' of Sm-KRAS/Lg-RBDα 10µL reaction across 1 plate, analysis
Figure 5E: Z' of Sm-KRAS/Lg-RBDα 20µL reaction across 1 plate
Figure 5E: Z' of Sm-KRAS/Lg-RBDα 20µL reaction across 1 plate, analysis
Figure 5F–G:
  Z' of Sm-KRAS/Lg-RBDα 10 and 20µL reaction across 10 plates, Plate 1
  Z' of Sm-KRAS/Lg-RBDα 10 and 20µL reaction across 10 plates, Plate 2
  Z' of Sm-KRAS/Lg-RBDα 10 and 20µL reaction across 10 plates, Plate 3
  Z' of Sm-KRAS/Lg-RBDα 10 and 20µL reaction across 10 plates, Plate 4
  Z' of Sm-KRAS/Lg-RBDα 10 and 20µL reaction across 10 plates, Plate 5
  Z' of Sm-KRAS/Lg-RBDα 10 and 20µL reaction across 10 plates, Plate 6
  Z' of Sm-KRAS/Lg-RBDα 10 and 20µL reaction across 10 plates, Plate 7
  Z' of Sm-KRAS/Lg-RBDα 10 and 20µL reaction across 10 plates, Plate 8
  Z' of Sm-KRAS/Lg-RBDα 10 and 20µL reaction across 10 plates, Plate 9
  Z' of Sm-KRAS/Lg-RBDα 10 and 20µL reaction across 10 plates, Plate 10
  Z' of Sm-KRAS/Lg-RBDα 10 and 20µL reaction across 10 plates, analysis
Figure 6B: CHO expression of Lg-KRAS/Sm-RAF
Figure 6B: CHO expression of Lg-KRAS/Sm-RAF, analysis
Figure 6C: ARS-1620 treatment on CHO expression of Lg-RAS/Sm-RAF
Figure 6C: ARS-1620 treatment on CHO expression of Lg-RAS/Sm-RAF, analysis
Figure 6D:
  Z' of CHO expressed Lg-KRAS/Sm-RAF 10µL reaction, Plate 1
  Z' of CHO expressed Lg-KRAS/Sm-RAF 10µL reaction, Plate 2
  Z' of CHO expressed Lg-KRAS/Sm-RAF 10µL reaction, Plate 3
  Z' of CHO expressed Lg-KRAS/Sm-RAF 10µL reaction, Plate 4
  Z' of CHO expressed Lg-KRAS/Sm-RAF 10µL reaction, Plate 5
  Z' of CHO expressed Lg-KRAS/Sm-RAF 10µL reaction, Plate 6
  Z' of CHO expressed Lg-KRAS/Sm-RAF 10µL reaction, Plate 7
  Z' of CHO expressed Lg-KRAS/Sm-RAF 10µL reaction, Plate 8
  Z' of CHO expressed Lg-KRAS/Sm-RAF 10µL reaction, analysis
**•   Figure 2–Figure 6 analysis (all CSV):**

In all figures, we presented the normalised or the relative luminescence/Fluorescence data of each experiment. 

Data are available under the terms of the Creative Commons Attribution 4.0 International license (CC-BY 4.0). 

## References

[1] Degorce F Card A Soh S: HTRF: A technology tailored for drug discovery - a review of theoretical aspects and recent applications. *Curr Chem Genomics.* 2009;3:22–32. 10.2174/1875397300903010022 20161833 PMC2802762

[2] Janzen WP: Screening technologies for small molecule discovery: the state of the art. *Chem Biol.* 2014;21(9):1162–1170. 10.1016/j.chembiol.2014.07.015 25237860

[3] Hancock JF: Ras proteins: different signals from different locations. *Nat Rev Mol Cell Biol.* 2003;4(5):373–384. 10.1038/nrm1105 12728271

[4] Killoran RC Smith MJ: Conformational resolution of nucleotide cycling and effector interactions for multiple small GTPases determined in parallel. *J Biol Chem.* 2019;294(25):9937–9948. 10.1074/jbc.RA119.008653 31088913 PMC6597838

[5] Knickelbein K Zhang L: Mutant KRAS as a critical determinant of the therapeutic response of colorectal cancer. *Genes Dis.* 2015;2(1):4–12. 10.1016/j.gendis.2014.10.002 25815366 PMC4372129

[6] McCormick F: Progress in targeting RAS with small molecule drugs. *Biochem J.* 2019;476(2):365–374. 10.1042/BCJ20170441 30705085

[7] Stephen AG Esposito D Bagni RK: Dragging ras back in the ring. *Cancer Cell.* 2014;25(3):272–281. 10.1016/j.ccr.2014.02.017 24651010

[8] Li S Balmain A Counter CM: A model for RAS mutation patterns in cancers: finding the sweet spot. *Nat Rev Cancer.* 2018;18(12):767–777. 10.1038/s41568-018-0076-6 30420765

[9] Simanshu DK Nissley DV McCormick F: RAS Proteins and Their Regulators in Human Disease. *Cell.* 2017;170(1):17–33. 10.1016/j.cell.2017.06.009 28666118 PMC5555610

[10] Bivona TG: Dampening oncogenic RAS signaling. *Science.* 2019;363(6433):1280–1281. 10.1126/science.aav6703 30898918

[11] Castellano E Sheridan C Thin MZ: Requirement for interaction of PI3-kinase p110α with RAS in lung tumor maintenance. *Cancer Cell.* 2013;24(5):617–630. 10.1016/j.ccr.2013.09.012 24229709 PMC3826036

[12] Gupta S Ramjaun AR Haiko P: Binding of ras to phosphoinositide 3-kinase p110alpha is required for ras-driven tumorigenesis in mice. *Cell.* 2007;129(5):957–968. 10.1016/j.cell.2007.03.051 17540175

[13] , , Fischer A Hekman M Kuhlmann J: B- and C-RAF display essential differences in their binding to Ras: the isotype-specific N terminus of B-RAF facilitates Ras binding. *J Biol Chem.* 2007;282(36):26503–26516. 10.1074/jbc.M607458200 17635919

[14] , , Fritsch R de Krijger I Fritsch K: RAS and RHO families of GTPases directly regulate distinct phosphoinositide 3-kinase isoforms. *Cell.* 2013;153(5):1050–1063. 10.1016/j.cell.2013.04.031 23706742 PMC3690480

[15] Pacold ME Suire S Perisic O: Crystal structure and functional analysis of Ras binding to its effector phosphoinositide 3-kinase gamma. *Cell.* 2000;103(6):931–943. 10.1016/s0092-8674(00)00196-3 11136978

[16] Dixon AS Schwinn MK Hall MP: NanoLuc Complementation Reporter Optimized for Accurate Measurement of Protein Interactions in Cells. *ACS Chem Biol.* 2016;11(2):400–408. 10.1021/acschembio.5b00753 26569370

[17] Johnston PA Johnston PA: Cellular platforms for HTS: three case studies. *Drug Discov Today.* 2002;7(6):353–363. 10.1016/s1359-6446(01)02140-7 11893544

[18] Wang Z Wang X He Z: [Establishment of drug screening assay and pharmacodynamic evaluation method targeting influenza RNA polymerase]. *Yao Xue Xue Bao.* 2012;47(9):1159–1163. 23227545

[19] Ismail M: NBBA (NanoBit Biochemical Assay).2020 10.17605/OSF.IO/MGKQV

[20] Winter JJ Anderson M Blades K: Small molecule binding sites on the Ras:SOS complex can be exploited for inhibition of Ras activation. *J Med Chem.* 2015;58(5):2265–2274. 10.1021/jm501660t 25695162

[21] Athuluri-Divakar SK Vasquez-Del Carpio R Dutta K: A Small Molecule RAS-Mimetic Disrupts RAS Association with Effector Proteins to Block Signaling. *Cell.* 2016;165(3):643–655. 10.1016/j.cell.2016.03.045 27104980 PMC5006944

[22] Janes MR Zhang J Li LS: Targeting KRAS Mutant Cancers with a Covalent G12C-Specific Inhibitor. *Cell.* 2018;172(3):578–589 e517. 10.1016/j.cell.2018.01.006 29373830

[23] Welsch ME Kaplan A Chambers JM: Multivalent Small-Molecule Pan-RAS Inhibitors. *Cell.* 2017;168(5):878–889 e829. 10.1016/j.cell.2017.02.006 28235199 PMC5362268

[24] Kessler D Gmachl M Mantoulidis A: Drugging an undruggable pocket on KRAS. *Proc Natl Acad Sci U S A.* 2019;116(32):15823–15829. 10.1073/pnas.1904529116 31332011 PMC6689897

[25] Zhang JH Chung TD Oldenburg KR: A Simple Statistical Parameter for Use in Evaluation and Validation of High Throughput Screening Assays. *J Biomol Screen.* 1999;4(2):67–73. 10.1177/108705719900400206 10838414

[26] Fabian JR Vojtek AB Cooper JA: A single amino acid change in Raf-1 inhibits Ras binding and alters Raf-1 function. *Proc Natl Acad Sci U S A.* 1994;91(13):5982–5986. 10.1073/pnas.91.13.5982 8016101 PMC44121

