# Supplementary material for: Development of a cell-free split-luciferase biochemical assay as a tool for screening for inhibitors of challenging protein-protein interaction targets
Source: Wellcome Open Res. 2020 Feb 6;5:20. [Version 1] doi: 10.12688/wellcomeopenres.15675.1 (PMC7308888; doi:10.12688/wellcomeopenres.15675.1)
Supplement: Peer review history

# Peer review: Reviewer response for version 1

**DOI:** 10.21956/wellcomeopenres.17179.r38408

**Published:** 2020-04-22

**Authors:** Nicolas Bery

**Affiliations:** 1 Cancer Research Centre of Toulouse, INSERM, Université Toulouse III Paul Sabatier, CNRS, Toulouse, France

Cooley *et al*. have implemented a biochemical assay from a cell-based split luciferase assay to monitor RAS protein-protein interaction (PPI) and RAS PPI inhibition. They show the development and use of this cell free assay as method to detect RAS PPI inhibition by small molecule inhibitors (three published RAS inhibitors) and one macromolecule (CRAF RBD). This study is well described, with the appropriate controls and the data presented supports their conclusions. The main advantage of this method is its potential application to high throughput screening (HTS) of small molecule inhibitors of PPI and this approach could be extended to any mutant RAS/effector interaction of choice. Overall, the methods provided in the manuscript give enough details to replicate the experiments but some information is missing. The authors should address these minor points:

1. Usually optimisation steps are needed for these kind of PPI probes (e.g. linker length and probes orientation). However, no information is provided concerning the optimisation procedures that were employed to get the KRAS/effectors NanoBiT probes with the exception of the orientation of the SmBiT/LgBiT molecules on CRAF full-length and KRAS ^G12C^. Therefore, it is unclear whether the NanoBiT KRAS/effector probes described in this study can be further optimised. This is important information to know for potential future users:
  - It should be indicated whether the linker length between the Lg/SmBiT and KRAS/effectors was optimised. Their sequence should be added in the methods section.
  - All the RBDs (CRAF and PI3Ks) are tagged on their amino terminal end with a NanoBiT probe (Sm/LgBiT). It should be explained whether the opposite tagging combination (on the carboxy terminal end of the RBDs) was tested and compared to N terminal end tagging. For example, the structural data available for HRAS/CRAF RBD (PDB ID 4G3X, 4G0N) show that the N terminal end of HRAS is closer to the C terminal end of CRAF RBD, which suggests that cloning the Sm/LgBiT on the C terminal end of this RBD might give an optimal luciferase complementation and a higher luminescence signal.
2. The expression of the NanoBiT probes in the cell lysates should be controlled by Western blotting.
3. Additional information should be provided regarding the incubation time of the inhibitors in the cell lysates used in this study (20 minutes): how was this time chosen? Was it optimised?
4. In the methods section, the details of BI-2852 inhibitor treatment is not indicated (including the incubation time), the missing information should be added.
5. Figure 3: panels H/I: the authors should indicate in the figure legend what the dotted lines represent in these panels.

Is the rationale for developing the new method (or application) clearly explained?

Yes

Is the description of the method technically sound?

Yes

Are the conclusions about the method and its performance adequately supported by the findings presented in the article?

Yes

If any results are presented, are all the source data underlying the results available to ensure full reproducibility?

Yes

Are sufficient details provided to allow replication of the method development and its use by others?

Partly

Reviewer Expertise:

Development of cell-based assays to monitor PPI, targeting PPI with inhibitors (small molecules and macromolecules).

I confirm that I have read this submission and believe that I have an appropriate level of expertise to confirm that it is of an acceptable scientific standard, however I have significant reservations, as outlined above.

# Response

**Published:** 2020-05-14

**Authors:** Julian Downward

**Affiliations:** Crick Institute

**Authors' Response to Reviewer Report from Nicolas Bery**

**Point 1:**

   With regard to the linker length and sequence, in the case of N terminal, Lg-KRAS-G12C and Sm-RAF in pBiT1.1-N [TK/LgBiT] and pBiT2.1-N [TK/SmBiT] vectors respectively, these vectors were provided by Promega for our initial studies to test the assay. Both vectors (either N terminal Sm or Lg-BiT) contained the same exact linker sequence encoding 13 amino acids  (GGGGSGGGGSAIA). They were not further optimised. However, in the case of the BiBiT vectors pBiT2.3-N [CMV/SmBiT/Blast] Vector (pRSG199) or pBiT1.3-N [CMV/LgBiT/Hyg] Vector (pRSG197) that we used for the rest of our studies, the restriction sites SacI and BamHI were introduced to provide an N terminal linker of 18 amino acids (GSSGGGGSGGGGSSGGAQ), 5 amino acids longer than that in the other vectors.  Nevertheless, we did see a good signal arising from the RAS-G12C and the p110-RBDs, so we did not apply any further optimisation to the linker length or sequence. The linker sequences have been added in the paper.

   As regards the orientation of the tags, the initial vectors provided by Promega expressing N terminal Lg-KRAS and N terminal Sm-CRAF produced a strong signal, indicating that placing the tags at the N termini of both proteins did not block or prevent the production of a strong luminescence signal that indicates the RAS/CRAF interaction. Therefore, we employed the same orientation of the tags on the BiBiT vectors for Sm-KRAS G12C and p110-RBDs. Further, in the case of the p110-RBDs, due to the fact that they are not very soluble, we also considered placing the Lg-BiT on the N terminus to ensure that it can provide maximum solubility to the RBDs. We managed to produce a significant signal representing the interaction of RAS and the p110-RBDs. Therefore, we did not try different orientations for the tag in the case of the p110-RBDs. In the case of RAS, we considered it to be important to have the tag on the N terminus and not the C terminus to avoid any prevention of RAS localisation to the cellular membrane. Although this was our rationale for the tag orientations for our study with these target proteins, we would recommend that other studies with different protein targets should test all tag orientation combinations.

**Point 2:**

   Due to the lack of an efficient antibody for the Lg-BiT we used antibodies against KRAS, CRAF and p110α to detect the transiently expressed proteins. We added a new figure in the paper (Figure 7) where we show an increase in the signal intensity and/or increased molecular weight (due to the addition of the Sm or the Lg-BiT tags) between the transiently expressed and the endogenous proteins.

**Point 3:**

   The 20 minute incubation was chosen to ensure full target engagement of inhibitors with RAS. As one of the inhibitors used in the study (Pan RAS 3144) is a weak binder, we wanted to give the maximum opportunity for the inhibitor to bind to RAS. We tested both 20 and 30min but saw no difference by increasing the time to 30min. Therefore we added the 20min time-point. However we recommend for other inhibitors and protein targets that shorter or longer incubation times should be tested. This has been added in the materials and methods section.

**Point 4:**

   The BI-2852 information has been added to the methods section. The same treatment and experimental condition was used as for the Pan RAS 3144 inhibitor.

**Point 5:**

   The figure legend of Figure 3H, I has been corrected to describe the dotted lines.

# Peer review: Reviewer response for version 1

**DOI:** 10.21956/wellcomeopenres.17179.r37848

**Published:** 2020-02-17

**Authors:** Andrew G. Stephen

**Affiliations:** 1 NCI RAS Initiative, Cancer Research Technology Program, Frederick National Laboratory for Cancer Research, Frederick, MD, USA

Cooley and co-authors have adapted a cell based split luciferase assay originally designed for intracellular protein:protein interactions into a biochemical assay using cellular lysates. The authors apply this approach to measuring the interaction between KRAS and PI3Ka, which has previously been characterized as a relatively weak interaction. One significant benefit of this approach could be the improved solubility of the proteins in the HEK292 cells, which the authors claim combination of the largeBiT fragment with PI3K appears to improve solubility (although they do not show any direct evidence of this). Optimal signals were obtained between KRAS and PI3Ka-RBD rather than full length PI3Ka. The assay is specific as PI3K mutants do not bind and CRAF-RBD effectively competes. Cooley *et al.* also show the interaction between PI3K-RBD and KRAS is inhibited by 3 previously characterized RAS inhibitors and provide data that supports the assay is amenable to small molecule screening in 384 well plates. The methods are described in enough detail that would allow replication by others and the authors outline the benefits/limitations of working with cell lysates rather than live cells. Overall the manuscript provides a new approach for interrogating the interaction between PI3K and RAS and can be adapted as an HTS compatible assay for other binding partners with low solubility or weak binding affinity. The following minor issues should be addressed:

1. While Cooley *et al.* were unable to use HTRF to detect the binding of recombinant KRAS and PI3Ka, Kessler *et al*. (2019) ^1^ published a method using alpha technology to detect their interaction and showed inhibition by BI2852. A reference should be made to this work and a brief comment made of the differences between the HTRF approach reported here and the alpha assay described by Kessler *et al*.
2. The authors clearly show that no specific interaction was observed between KRAS and PI3Ka irrespective of which protein was the acceptor or the donor. However, when KRAS was labeled with streptavidin-europium the fluorescence signal was ~10 ^5^ times larger than the KRAS-CRAF-RBD experiment or when PI3Ka was labeled with europium (compare Figure 2B with 2A or 2C). The statement in the text indicating “only a low signal was achieved (Figure 2B, C)” is confusing and the authors should clarify.

Is the rationale for developing the new method (or application) clearly explained?

Yes

Is the description of the method technically sound?

Yes

Are the conclusions about the method and its performance adequately supported by the findings presented in the article?

Yes

If any results are presented, are all the source data underlying the results available to ensure full reproducibility?

Yes

Are sufficient details provided to allow replication of the method development and its use by others?

Yes

Reviewer Expertise:

Biochemical and biophysical analysis of RAS and RAS effector interactions.

I confirm that I have read this submission and believe that I have an appropriate level of expertise to confirm that it is of an acceptable scientific standard, however I have significant reservations, as outlined above.

## References

[1] : Drugging an undruggable pocket on KRAS. *Proc Natl Acad Sci U S A*.2019;116(32) : 10.1073/pnas.1904529116 15823-15829 10.1073/pnas.1904529116 31332011PMC6689897

# Response

**Published:** 2020-04-02

**Authors:** Julian Downward

**Affiliations:** Crick Institute

We thank Andrew Stephen for his constructive comments. In response to the specific points, we now reference the paper by Kessler et al. (2019) describing the use of alpha technology to detect the interaction of KRAS and PI3Ka and show its inhibition by BI2852. Compared to our HTRF assay, the alpha assay was run using lower KRAS concentrations (10nM versus 3µM), but similar PI3Ka levels (10nM). We have clarified the statement about the level of  fluorescence signal in Figure 2B: the signal strength was high, but not GTP dependent, so considered to be non-specific.

# Response

**Published:** 2020-05-14

**Authors:** Julian Downward

**Affiliations:** Crick Institute

**Authors’ Response to Reviewer Report by Andrew Stephens**

   We thank Stephens for his comments. In response to the specific points, we now reference the paper by Kessler et al. (2019) describing the use of alpha technology to detect the interaction of KRAS and PI3Ka and show its inhibition by BI2852. Compared to our HTRF assay, the alpha assay was run using lower KRAS concentrations (10nM versus 3µM), but similar PI3Ka levels (10nM). We have clarified the statement about the level of  fluorescence signal in Figure 2B: the signal strength was high, but not GTP dependent, so considered to be non-specific.
